# *CAV1* rs7804372 (T29107A) polymorphism might be a potential risk for digestive cancers: A protocol for systematic review and meta analysis

**DOI:** 10.1097/MD.0000000000026186

**Published:** 2021-06-18

**Authors:** Pei Chen, Yu-Ling Zhang, Bai Xue, Ji-Ru Wang

**Affiliations:** a Department of Basic Medicine; b Department of Medical Technology, Jiangsu College of Nursing, Huai an, Jiangsu, China.

**Keywords:** CAV1, digestive cancers, meta-analysis, polymorphism

## Abstract

**Background:**

Caveolin-1 (CAV1) is an essential structural component of caveolae, regulates cellular processes through complex cellular signaling pathways, and influences tumorigenicity. However, the role of the *CAV1* (rs7804372) polymorphism in digestive cancers remains inconclusive. The meta-analysis was performed to evaluate the effect of CAV1 polymorphism on digestive cancer susceptibility and to provide a basis for precise treatment.

**Methods:**

The databases of PubMed, EMBASE, Google Scholar and CNKI were used to retrieve the published studies on *CAV1* (rs7804372) polymorphism and susceptibility to digestive cancers up to June 2020. Two researchers conducted study screening, data extraction, and methodological quality evaluation separately according to inclusion and exclusion criteria. Review Manager 5.3 software was used to conduct the meta-analysis.

**Results:**

Six case-control studies were enrolled, including 2477 patients with digestive cancers and 2477 healthy controls. The pooled results showed that the *CAV1* rs7804372 (T29107A) polymorphism increased the risk of digestive cancer occurrence in the allele (*T* vs. *A*: odds ratio (OR) 1.33, 95% confidence interval (CI): 1.15–1.53, *P* < .01), homozygous (*TT* vs. *AA*: OR 1.72, 95% CI: 1.31–2.26, *P* < .01), heterozygous (*TA* vs. *AA*: OR 1.47, 95% CI: 1.21–1.78, *P* < .01), dominant (*TT* vs. *TA* + *AA*: OR 1.32, 95% CI: 1.18–1.48, *P* < .01), and recessive comparing models (*TT* + *TA* vs. *AA*: OR 1.61, 95% CI: 1.26–2.07, *P* < .01).

**Conclusion:**

Our results indicate that the *CAV1* (rs7804372) polymorphism may modify the occurrence of digestive cancers, and the presence of *T* allele or *TT* genotype of the *CAV1* (rs7804372) may increase the risk of digestive cancers.

## 1 Introduction

The incidence of malignant cancers is increasing every year with the development of global aging and unhealthy lifestyle. Globally, the incidence of digestive malignant tumors ranks among the forefront of cancer incidence.^[1]^ Digestive cancers, especially esophageal carcinoma, gastric cancer, colorectal cancer, and hepatocellular cancer, are a global pivotal epidemiological health concern. Compared with other cancers, patients with digestive cancers not only are required to receive comprehensive treatment such as surgery, radiotherapy and chemotherapy but also face adverse reactions, such as malnutrition, diarrhea, constipation caused by gastrointestinal dysfunction, as well as body function and self-image changes caused by artificial stoma. These problems significantly influence the prognosis of patients with digestive cancer.^[2]^

Caveolin-1 (CAV1) is the main membrane protein of caveolae, which are scaffolding proteins of plasma membrane invaginations.^[3]^ CAV1 is an essential structural component of caveolae, regulates cellular processes through complex cellular signaling pathways, and influences tumorigenicity. The structure and function of the caveolin-1 gene family are highly conserved in different species, indicating its essential role for maintaining cellular functions. CAV1 interacts with various signal transduction molecules through phosphorylation/dephosphorylation signaling.^[4]^ It plays an important role in cholesterol and lipid transport, membrane transport, signal transduction, and cell adhesion.^[5]^ Additionally, CAV1 is involved in the regulation of various signaling pathways, such as cell proliferation, differentiation, apoptosis, migration, and angiogenesis, and is associated with the occurrence, development, invasion, and metastasis of various tumors.^[6]^ The carcinogenic role of CAV1 has been identified in several tumors, suggesting CAV1 as a novel therapeutic target for tumors. Several studies have explored the relationship between CAV1 polymorphisms and susceptibility to breast cancer,^[7]^ esophageal carcinoma,^[8]^ colorectal cancer,^[9]^ gastric cancer,^[10,11]^ hepatocellular,^[12]^ and other cancers.^[13,14]^ However, the results of the *CAV1* rs7804372 (T29107A) polymorphism and susceptibility to digestive cancer remain controversial. Thus, this meta-analysis was conducted to verify the contribution of the *CAV1* rs7804372 (T29107A) polymorphism to digestive cancer susceptibility to provide evidence for precise clinical treatment.

## 2 Methods

Ethical review was not required since our manuscript is meta-analysis. This meta-analysis was conducted in comply with the guidelines for preferred reporting items for systematic reviews and meta-analyses (PRISMA statement).

### 2.1 Searching strategy and selection criteria

The literature related to the association of *CAV1* (rs7804372) polymorphism and digestive cancer susceptibility was searched online through the PubMed, EMBASE, Google Scholar, CNKI, Wan Fang and VIP databases from their inception up to June 2020. The following terms were used for retrieving valuable articles: [(CAV1) or (Caveolin-1) or (caveolin)] and [rs7804372 OR T29107A] [polymorphism or genotype or mutation or variant] and [oral or esophageal or gastric or hepatocellular or colorectal]. The references of retrieved articles were manually reviewed for identifying other qualified articles.

Studies were enrolled according to the following criteria:

1. all published studies must have explored the association between the *CAV1* rs7804372 (T29107A) polymorphism and digestive cancer;
2. case control studies;
3. genotype distribution in case and control can be directly acquired or calculated;
4. full text can be acquired directly.

The meta-analysis excluded studies according to the following criteria:

1. animal or cell line research;
2. systematic review, meta-analysis and repetitive reports;
3. unavailability of genotype data distribution.

### 2.2 Data extraction

Using a specified data extraction table, two investigators completed the study screening, data extraction, and sorted independently, discussed and negotiated the disputed parts, finally reached a consensus. Data extraction mainly includes the first author's name, publication year, sample size, tumor differentiation, clinical stage, and lymph node metastasis. The New Castle–Ottawa scale was used for assessing the quality of each study.

### 2.3 Statistical analyses

Review Manager 5.3 software was used for analyzing five genetic models of the *CAV1* gene polymorphism, including allele (*T* vs. *A*), homozygous (*TT* vs. *AA*), heterozygous (*TA* vs *AA*), dominant (*TT* vs. *TA* + *AA*), and recessive comparing models (*TT* + *TA* vs. *AA*), where *TT* is a homozygote of a wild-type allele, *TA* is a heterozygote, and *AA* is a homozygote of a mutant allele. The comparison between *CAV1* (rs7804372) polymorphism and digestive cancer was expressed using pooled odds ratio (OR) and 95% confidence interval (CI). The χ^2^ test and I^2^ statistics were used to judge whether there was heterogeneity among the studies; *P* >.10 and I^2^ < 50% could be considered that there was no statistical heterogeneity between the research results, and the fixed effect model was selected for data consolidation; *P* ≤ .10 and I^2^ ≥50% could be considered that there was statistical heterogeneity between the research results, and a random effect model was used for data consolidation. The Hardy–Weinberg equilibrium of the control group was evaluated using the χ^2^ test, and the expected and actual genotype frequencies of the control group were compared. In this meta-analysis, *P-*values of ≤.05 were considered statistically significant.

## 3 Results

### 3.1 Characteristics of enrolled studies

According to the inclusion and exclusion criteria, six case-control studies were included, including 2477 patients with digestive cancers and 2477 healthy controls. The positive rate of the T gene locus was 74.22% in patients and 68.21% in healthy controls (Tables 1 and 2).

**Table 1:** The main characteristics of included studies.

First author	Ethnicity	Cancer type	Case / control	Test method
Zhang 2014	Asian	Gastric cancer	412/412	Spectrometry
Lin 2014	Asian	Gastric cancer	358/358	RT-PCR
Wang 2014	Asian	Esophageal cancer	427/427	RT-PCR
Hsu 2013	Asian	Hepatocellular cancer	298/298	RT-PCR
Bau 2011	Asian	Oral cancer	620/620	RT-PCR
Yang 2010	Asian	Colorectal cancer	363/362	RT-PCR

**Table 2:** Allele frequency and genotype distribution of control and case in included studies.

		Control					Case				
First Author	n	TT	AT	AA	T	A	TT	AT	AA	T	A
Zhang 2014	824	210	136	66	556	268	239	134	39	612	212
Lin 2014	756	192	133	33	517	199	188	135	35	511	205
Wang 2014	854	221	166	40	608	246	259	143	25	661	193
Hsu 2013	596	152	98	48	402	194	166	93	39	425	171
Bau 2011	1240	306	206	108	818	422	363	193	64	919	321
Yang 2010	724	179	120	63	478	246	216	117	29	549	175
Total	4954	1260	859	358	3379	1575	1431	815	231	3677	1277

### 3.2 Test of heterogeneity

χ^2^ test and I^2^ statistics were used for assessing the heterogeneity of the enrolled studies. Results indicate that there was statistical heterogeneity in the heterozygous comparison model (*TA* vs *AA*) and dominant (*TT* vs *TA* + *AA*), the fixed effect model was used for evaluating the pooled OR and 95% CI for those comparisons. No statistical heterogeneity was discovered in the allele (*T* vs *A*), homozygous comparison models (*TT* vs *AA*) and recessive comparison models (*TT* + *TA* vs *AA*), the random effect model was used for evaluating the pooled OR and 95% CI for those comparisons.

### 3.3 Sensitivity analysis and publication bias

**Figure 1:**
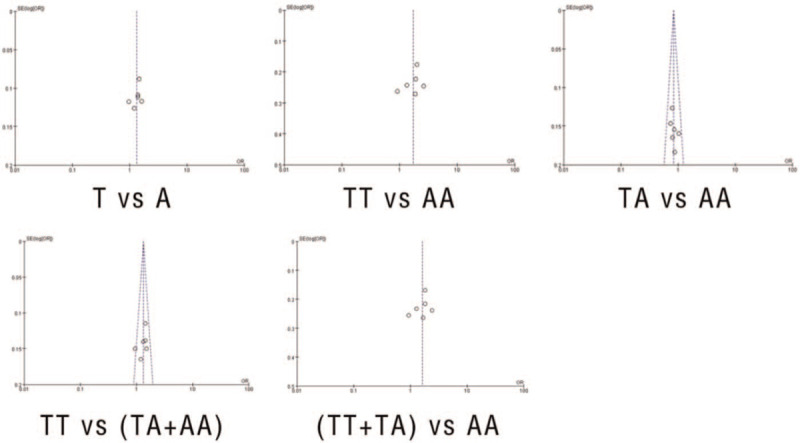
Funnel plot for publication bias. A funnel chart was used for evaluating publication bias of the studies. The shape of the funnel chart did not show any evidence of significant asymmetry in the dominant model.

A sensitivity analysis was performed for assessing the influence of each study on the overall result by eliminating every study step by step individually. The results suggested that there were no independent studies that significantly influenced the pooled ORs. Funnel charts were used for evaluating publication bias. The funnel chart did not show any significant asymmetry (Figure 1).

### 3.4 Meta-analysis of CAV1 (rs7804372) polymorphism and digestive cancer susceptibility

**Figure 2:**
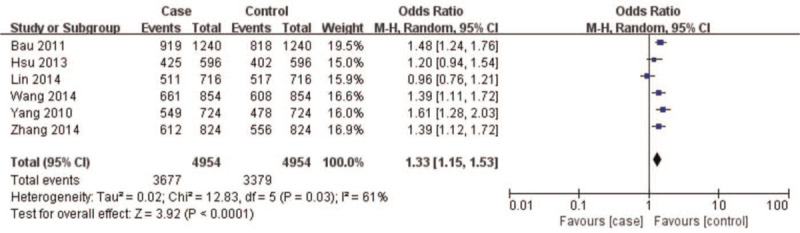
Forest plot of allele *T* vs *A* genotype. Overall pooled results revealed an increased risk of the *CAV1* (rs7804372) polymorphism for digestive cancer in the allele comparing model (*T vs A*: odds ratio 1.33, 95% confidence interval: 1.15–1.53, *P* < .01), and heterogeneity among the included studies was observed (I^2^ = 61%, *P* = .03). Results showed that the risk of *CAV1* (rs7804372) polymorphism *T* allele carriers was 1.33 times higher than that of *A* allele carriers.

**Figure 3:**
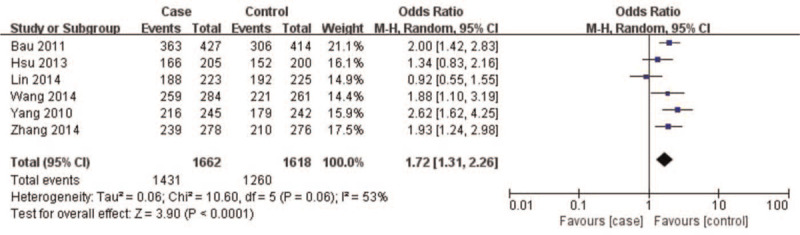
Forest plot of *TT* vs *AA* genotypes. Overall pooled results revealed an increased risk of the *CAV1* (rs7804372) polymorphism for digestive cancer in the homozygous comparison model (*TT* vs *AA*: odds ratio 1.72, 95% confidence interval: 1.31–2.26, *P* < .01). Heterogeneity among the included studies was observed (I^2^ = 53%, *P* = .06). Results showed that the risk of *CAV1* (rs7804372) polymorphism *TT* genotype carriers was 1.72 times higher than that of *AA* genotype carriers.

**Figure 4:**
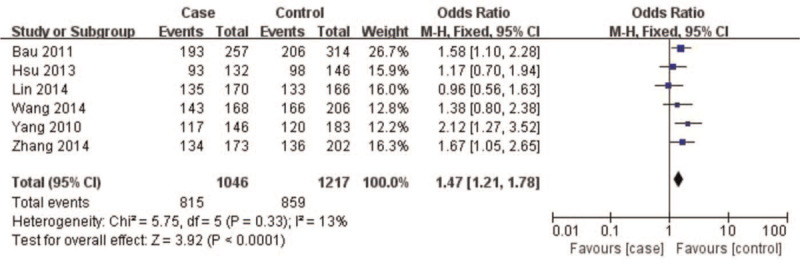
Forest plot of *TA vs AA* genotypes. Overall pooled results revealed an increased risk of *CAV1* (rs7804372) polymorphism for digestive cancer in a heterozygous comparison model (*TA* vs *AA*: odds ratio 1.47, 95% confidence interval: 1.21–1.78, *P* < .01). No heterogeneity among the included studies was observed (I^2^ = 13%, *P* = .33). Results showed that the risk of *CAV1* (rs7804372) polymorphism *TA* genotype carriers was 1.47 times higher than that of *AA* genotype carriers.

**Figure 5:**
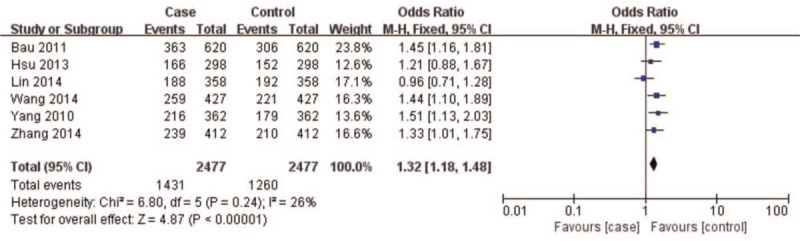
Forest plot of *TT vs* (*TA* + *AA*) genotypes. Overall pooled results revealed an increased risk of the *CAV1* (rs7804372) polymorphism for digestive cancer in the dominant comparison model (*TT* vs *TA* + *AA*: odds ratio 1.32, 95% confidence interval: 1.18–1.48, *P* < .01). Heterogeneity among the included studies was observed (*I*^*2*^ = 26%, *P* = 0.24). Results showed that the risk of *CAV1* (rs7804372) polymorphism *TT* genotype carriers was 1.31 times higher than that of *(TA+AA)* genotype carriers.

**Figure 6:**
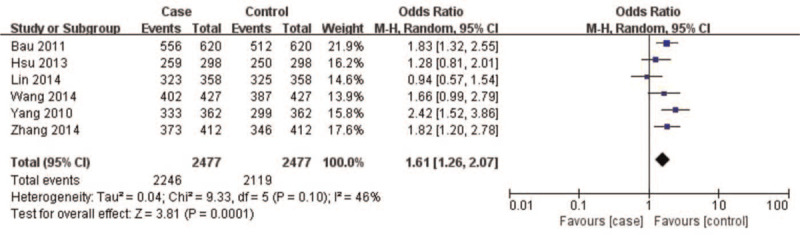
Forest plot of (*TT* + *TA*) vs *AA* genotypes. Overall pooled results revealed an increased risk of the *CAV1* (rs7804372) polymorphism for digestive cancer in a recessive comparison model (*TT* + *TA* vs *AA*: odds ratio 1.61, 95% confidence interval: 1.26–2.07, *P* < .01). No heterogeneity among the included studies was observed (I^2^ = 46%, *P* = .10). Results showed that the risk of *CAV1* (rs7804372) polymorphism *(TT* + *TA)* genotype carriers was 1.61 times higher than that of the *AA* genotype carriers.

A summary of meta-analysis findings regarding the relationship between the *CAV1* (rs7804372) polymorphism and digestive cancer is presented in (Table 3 and Figures 2–6) Overall pooled results revealed an increasing risk of *CAV1* (rs7804372) polymorphism in digestive cancer in the allele (*T* vs *A*: OR 1.33, 95% CI: 1.15–1.53, *P* < .01), homozygous (*TT* vs *AA*: OR 1.72, 95% CI: 1.31–2.26, *P* < .01), heterozygous (*TA* vs *AA*: OR 1.47, 95% CI: 1.21–1.78, *P* < .01), dominant (*TT* vs *TA* + *AA*: OR 1.32, 95% CI: 1.18–1.48, *P* < .01), and recessive comparison models (*TT* + *TA* vs *AA*: OR 1.61, 95% CI: 1.26–2.07, *P* < .01).

**Table 3:** Meta-analysis of Cav-1 rs7804372 gene polymorphism and tumor susceptibility of digestive system.

		Association test			Heterogeneity test		
Genotype	n	OR (95%CI)	*Z*	*P*	χ^2^	*P*	I^2^
T vs A	9908	1.33[1.15, 1.53]	3.92	<.01	12.83	<.01	61%
TT vs AA	3280	1.72[1.31, 2.26]	3.90	<.01	10.60	<.01	53%
TA vs AA	2263	1.47[1.21, 1.78]	3.92	<.01	5.75	.33	13%
TT vs (TA+AA)	4954	1.32[1.18, 1.48]	4.87	<.01	6.80	.24	26%
(TT+TA) vs AA	4954	1.61[1.26, 2.07]	3.81	<.01	9.33	.10	46%

## 4 Discussion

CAV1 is one of the critical components of the integral membrane protein that makes up caveolins. It has been reported to be associated with esophageal carcinoma,^[15,16]^ prostate cancer,^[17,18]^ colon cancer,^[19]^ breast cancer,^[20]^ bladder cancer,^[21]^ lung cancer,^[22]^ and others,^[23,24]^ which can promote or suppress tumor occurrence and development.

According to the results from BioMuta-single-nucleotide variations in the cancer database, 45 modified residues and two modified functional residues of the *CAV1* polymorphism have been recorded, and some of them have been reported to be correlated with digestive cancers.^[25]^

Yang et al^[9]^ first reported the correlation of *CAV1* (rs7804372) polymorphism with colorectal cancer. Their results indicated that *CAV1* (rs7804372) was related to a higher susceptibility to colorectal cancer, with joint effects with smoking status on colorectal cancer susceptibility, and the *A* allele of *CAV1* (rs7804372) polymorphism might act as a potential biomarker for the early diagnosis, prediction, and targets for cancer therapy. Zhang et al^[10]^ reported the correlation of *CAV1* (rs7804372) polymorphism with gastric cancer, and their results indicated that *CAV1* (rs7804372) polymorphisms increased gastric cancer susceptibility and risk. Patients receiving the *CAV1* (rs7804372) *TT* haplotype had a higher gastric cancer risk and susceptibility than those with *AT* or *AA* haplotypes. Bau et al^[13]^ reported that *CAV1* (rs7804372) was involved in oral cancer, the *A* allele of *CAV1* (rs7804372) played a protective role to prevent cancer occurrence and the T allele may be a risky factor. The *TT* genotype of *CAV1* (rs7804372) may be associated with risk for oral cancer. Moreover, Wang et al^[8]^ also reached the same conclusion for esophageal cancer. While different conclusions were obtained, Lin et al.^[11]^ reported that there was no correlation between *CAV1* (rs7804372) polymorphism and gastric cancer susceptibility. Hsu et al^[12]^ evaluated the relationships between six single nucleotide polymorphism of the *CAV1* gene and hepatocellular cancer risk in a Taiwanese population. No significant association between rs7804372 polymorphism and hepatocellular cancer was observed. Owing to the inconsistent results between the *CAV1* (rs7804372) polymorphism and digestive cancer susceptibility, our team comprehensively searched all published studies on the *CAV1* (rs7804372) polymorphism with digestive cancer and performed a meta-analysis for obtaining a comprehensive correlation between them.

In our study, six studies were finally enrolled. To provide a more detailed overview of the relationship between *CAV1* (rs7804372) polymorphism and digestive cancer, five genetic models were used. Our results indicated that the *CAV1* (rs7804372) polymorphism was involved in risk for digestive cancer and influences the susceptibility to digestive cancer. The *T* allele, *TT* genotype, and *TA* of *CAV1* (rs7804372) induces an increasing risk of digestive cancers. This means that the *CAV1* (rs7804372) polymorphism can be regarded as a potential biomarker for early diagnosis, development, and prognosis prediction of digestive cancer.

Because of the few reported studies on the relationship between *CAV1* (rs7804372) gene polymorphism and digestive system tumor, the limitations of database retrieval and data collection, the relationship between the *CAV1* (rs7804372) gene polymorphism and age, sex, and clinical and pathological characteristics were not analyzed. Further studies with larger samples are needed for confirming the relationship between *CAV1* (rs7804372) gene polymorphism and digestive system tumors.

In conclusion, we confirmed that *CAV1* (rs7804372) can act as a valuable genetic susceptibility marker for digestive cancer. The presence of the allele *T* of *CAV1* (rs7804372) has a higher risk of digestive cancer than that of the allele *A*. There is a potential value of targeting the allele *A* for early diagnosis, prognosis prediction, and therapy for digestive cancers.

## Author contributions

**Data curation:** Bai Xue, Ji -Ru Wang.

**Methodology:** Ji -Ru Wang.

**Project administration:** Yu-ling Zhang.

**Writing – original draft:** Pei Chen.

**Writing – review & editing:** Yu-ling Zhang.
